# Beneficial effects of flavonoids on animal models of atherosclerosis: A systematic review and meta-analysis

**DOI:** 10.1016/j.isci.2023.108337

**Published:** 2023-10-27

**Authors:** Gege Liao, Wanlu Liu, Yiming Dai, Xiangxiang Shi, Yang Liu, Dongye Li, Tongda Xu

**Affiliations:** 1 Institute of Cardiovascular Disease Research, Xuzhou Medical University, Xuzhou, China; 2 Department of Cardiology, the Affiliated Hospital of Xuzhou Medical University, Xuzhou, China

**Keywords:** Natural sciences, Biological sciences, Physiology

## Abstract

Atherosclerosis is the main cause of cardiovascular diseases that seriously endanger human health. The existing treatment drugs are effective, but they have some side effects. Accumulating evidence suggests that flavonoids have attracted wide attention due to their multiple cardioprotective effects and fewer side effects. PubMed, Web of Science database, Embase, and Cochrane Library were searched for studies evaluating the effects of flavonoids against atherosclerosis. 119 studies published from August 1954 to April 2023 were included. Random-effects models were performed for synthesis. Compared with the control group, flavonoids significantly reduced longitudinal and cross-sectional plaque area. The findings indicated that flavonoids significantly reduced the concentrations of serum TC, TG, and LDL-C and increased serum HDL-C concentrations. Besides, flavonoids reduced the levels of circulating pro-inflammatory factors, including TNF-α, IL-1β, and IL-6, and increased the serum IL-10 level. This study provides evidence for the potential cardiovascular benefits of flavonoids.

## Highlights

• It is aimed to evaluate the effects of flavonoids against atherosclerosis
• A systematic review and meta-analysis of 119 studies are conducted
• It was found that flavonoids exert anti-atherosclerotic effects in animal models

## Graphical abstract

**Figure:**
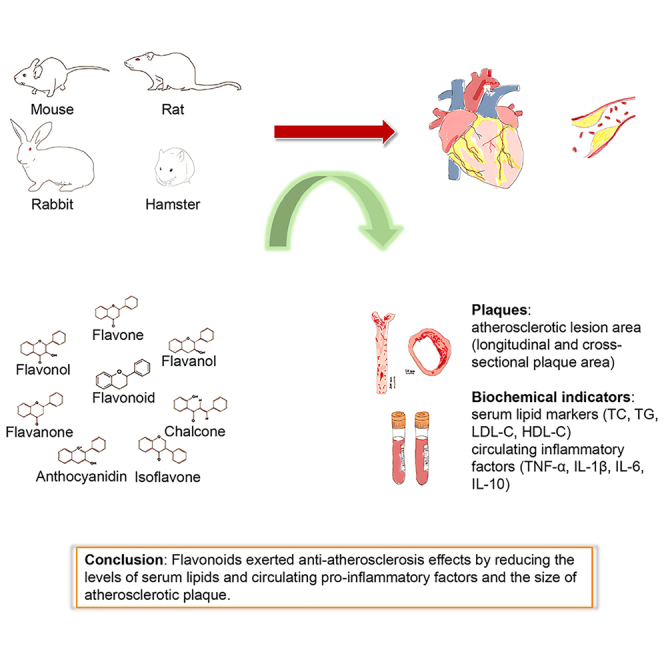

## Introduction

Atherosclerosis and subsequent coronary heart diseases (CHD) are major health concerns and the leading causes of morbidity and mortality worldwide.^1^ Anti-atherosclerosis studies to prevent the development of CHD have become a research hotspot. Atherosclerosis is a chronic inflammatory disease characterized by dysregulation of lipid metabolism and the formation of atherosclerotic plaques in the vessel wall.^2^^,^^3^ Atherosclerosis’s progression is drastically expedited by high levels of low-density lipoprotein cholesterol (LDL-C) and plaque rupture-induced thrombosis.^4^ In view of this, the primary strategies for preventing and treating atherosclerosis include lipid-lowering, anti-inflammation, anti-thrombosis, and re-establishment of arterial flow.^4^^,^^5^ The existing treatment drugs, including lipid-regulating drugs, anti-inflammation drugs, antiplatelet drugs, and thrombolytic anticoagulant drugs, are effective but inevitably have side effects such as bleeding and liver and kidney damage. Therefore, it is of great clinical significance to actively explore safe and effective therapeutic drugs for atherosclerosis.

In recent years, traditional Chinese medicine has been found to have multiple cardioprotective effects^6^ and has been widely used in the treatment of atherosclerosis in China and many other Asian countries.^7^ A growing body of studies has shown that flavonoids, as a kind of traditional Chinese medicine monomer, have an anti-atherosclerotic effect with a few side effects.^8^ Flavonoids, which are widely distributed in vegetables, fruits, tea, and other plants, are structurally characterized by a 15-carbon skeleton (C6-C3-C6).^9^^,^^10^^,^^11^ Based upon structural differences, flavonoids can be generally classified into seven major subclasses: flavonols, flavones, flavanols (flavan-3-ols or catechins), flavanones, anthocyanidins, isoflavones, and chalcones.^12^^,^^13^ Studies have reported that flavonoids have a variety of biological activities, including anti-cancer, anti-inflammatory, cardiac protection, neuroprotection, etc.^13^^,^^14^^,^^15^ The result of a multi-center randomized controlled trial suggested that flavonoids might exert anti-atherosclerosis effects through increasing high-density lipoprotein cholesterol (HDL-C) and apolipoprotein AI (Apo-AI) levels in patients with hyperlipidemia.^16^ A number of clinical studies have shown that flavonoids could prevent atherosclerosis in healthy volunteers through anti-inflammatory and anti-oxidative stress.^17^^,^^18^^,^^19^^,^^20^^,^^21^ A single-center randomized controlled trial found that flavonoids suppressed the growth of existing atherosclerotic plaques by 1.5-fold in postmenopausal women after 12-month follow-up.^22^ To date, clinical trials on flavonoids for the prevention and treatment of atherosclerosis are still scarce, and the specific mechanisms of anti-atherosclerosis of flavonoids are very shallow in clinical researches. Therefore, it is necessary to synthesize preclinical studies to refine the efficacy and intervention mechanisms of flavonoids against atherosclerosis.

Herein, we conducted a systematic review and meta-analysis of data from studies to appraise the efficacy of flavonoids on animal models of atherosclerosis. The changes in atherosclerotic lesion area, serum lipid markers, and circulating inflammatory factors were included as observation parameters. Furthermore, we summarized the currently known possible mechanisms of flavonoids in the treatment of atherosclerosis.

## Results

### Study selection

**Figure 1:**
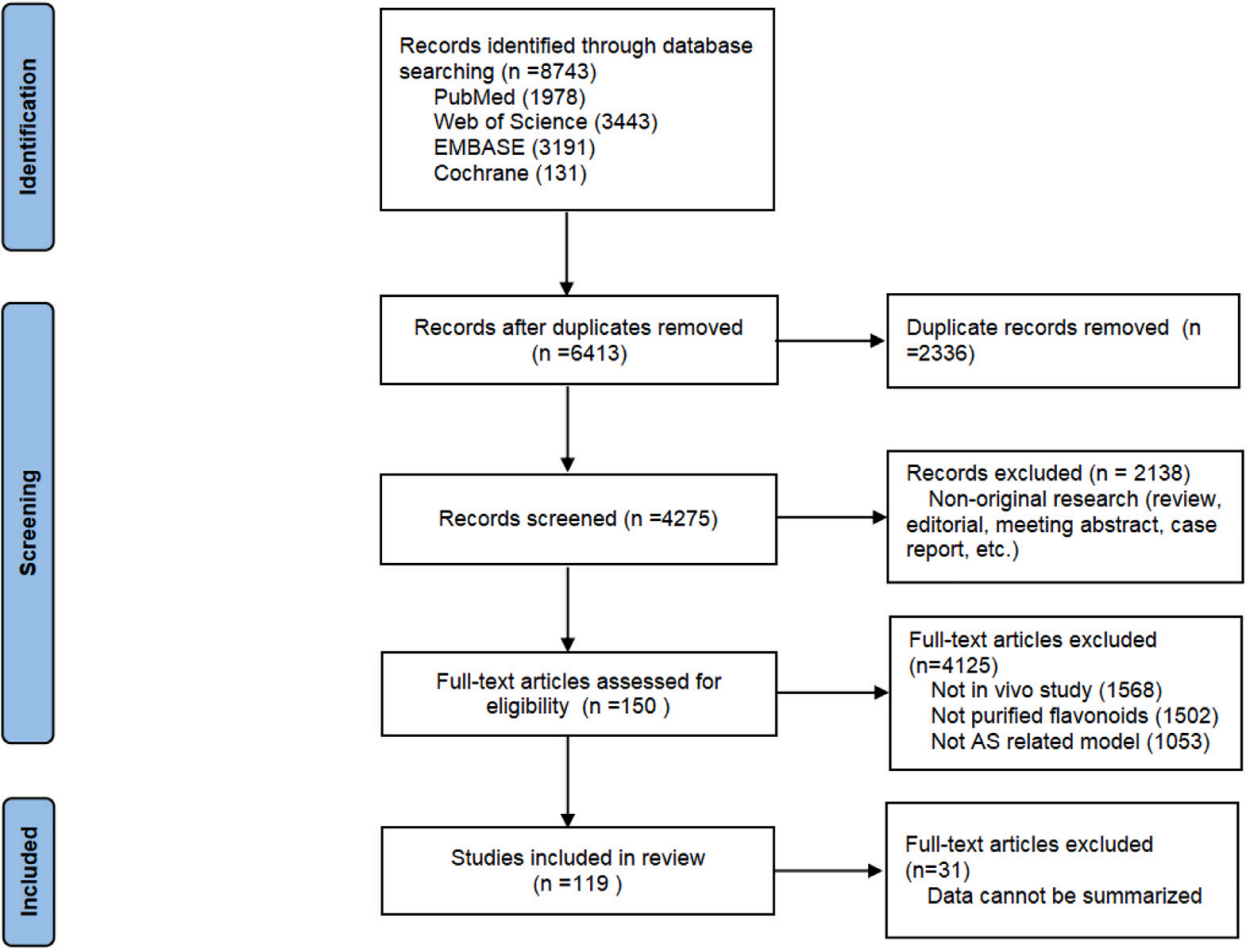
Flowchart of the search process

First, we systematically searched 8743 studies, of which 2336 duplications were removed. Then, we deleted 2138 studies by browsing the publication types. Next, based on the predefined exclusion criteria, 4125 articles were eliminated. Finally, after full-text evaluation, 31 studies were excluded, and 119 qualified articles were included in this meta-analysis. A flowchart depicting the process of selection is shown in Figure 1.

### Study characteristics

A total of 119 articles^23^^,^^24^^,^^25^^,^^26^^,^^27^^,^^28^^,^^29^^,^^30^^,^^31^^,^^32^^,^^33^^,^^34^^,^^35^^,^^36^^,^^37^^,^^38^^,^^39^^,^^40^^,^^41^^,^^42^^,^^43^^,^^44^^,^^45^^,^^46^^,^^47^^,^^48^^,^^49^^,^^50^^,^^51^^,^^52^^,^^53^^,^^54^^,^^55^^,^^56^^,^^57^^,^^58^^,^^59^^,^^60^^,^^61^^,^^62^^,^^63^^,^^64^^,^^65^^,^^66^^,^^67^^,^^68^^,^^69^^,^^70^^,^^71^^,^^72^^,^^73^^,^^74^^,^^75^^,^^76^^,^^77^^,^^78^^,^^79^^,^^80^^,^^81^^,^^82^^,^^83^^,^^84^^,^^85^^,^^86^^,^^87^^,^^88^^,^^89^^,^^90^^,^^91^^,^^92^^,^^93^^,^^94^^,^^95^^,^^96^^,^^97^^,^^98^^,^^99^^,^^100^^,^^101^^,^^102^^,^^103^^,^^104^^,^^105^^,^^106^^,^^107^^,^^108^^,^^109^^,^^110^^,^^111^^,^^112^^,^^113^^,^^114^^,^^115^^,^^116^^,^^117^^,^^118^^,^^119^^,^^120^^,^^121^^,^^122^^,^^123^^,^^124^^,^^125^^,^^126^^,^^127^^,^^128^^,^^129^^,^^130^^,^^131^^,^^132^^,^^133^^,^^134^^,^^135^^,^^136^^,^^137^^,^^138^^,^^139^^,^^140^^,^^141^ investigated the effects of 7 subclasses of flavonoids on atherosclerotic animal models. One study^23^ used two types of genetically modified mice. Three articles^24^^,^^52^^,^^98^ verified the efficacy of two different flavonoids. One^111^ verified the efficacy of four different flavonoids. Thus, there were actually 126 paired experiments. Of these 126 paired studies, 81 investigations^23^^,^^25^^,^^26^^,^^28^^,^^30^^,^^31^^,^^33^^,^^35^^,^^39^^,^^42^^,^^47^^,^^54^^,^^55^^,^^56^^,^^57^^,^^58^^,^^60^^,^^61^^,^^62^^,^^63^^,^^64^^,^^65^^,^^66^^,^^67^^,^^68^^,^^141^ had mice as model animals, 24 studies^27^^,^^36^^,^^37^^,^^40^^,^^43^^,^^49^^,^^50^^,^^51^^,^^72^^,^^83^^,^^86^^,^^87^^,^^88^^,^^89^^,^^141^ had rats as subjects, 15 experiments^29^^,^^32^^,^^38^^,^^41^^,^^44^^,^^48^^,^^52^^,^^53^^,^^59^^,^^78^^,^^95^^,^^109^^,^^129^^,^^130^ had rabbits as research objects, and 6 researches^24^^,^^34^^,^^45^^,^^46^^,^^118^ had hamsters as laboratory animals. The gender, age, and weight of the experimental animals varied from study to study. The administration route, dosage, and duration of flavonoids were also different in disparate publications. The basic characteristics of the individual studies are tabulated in Table S1.

### Study quality

**Figure 2:**
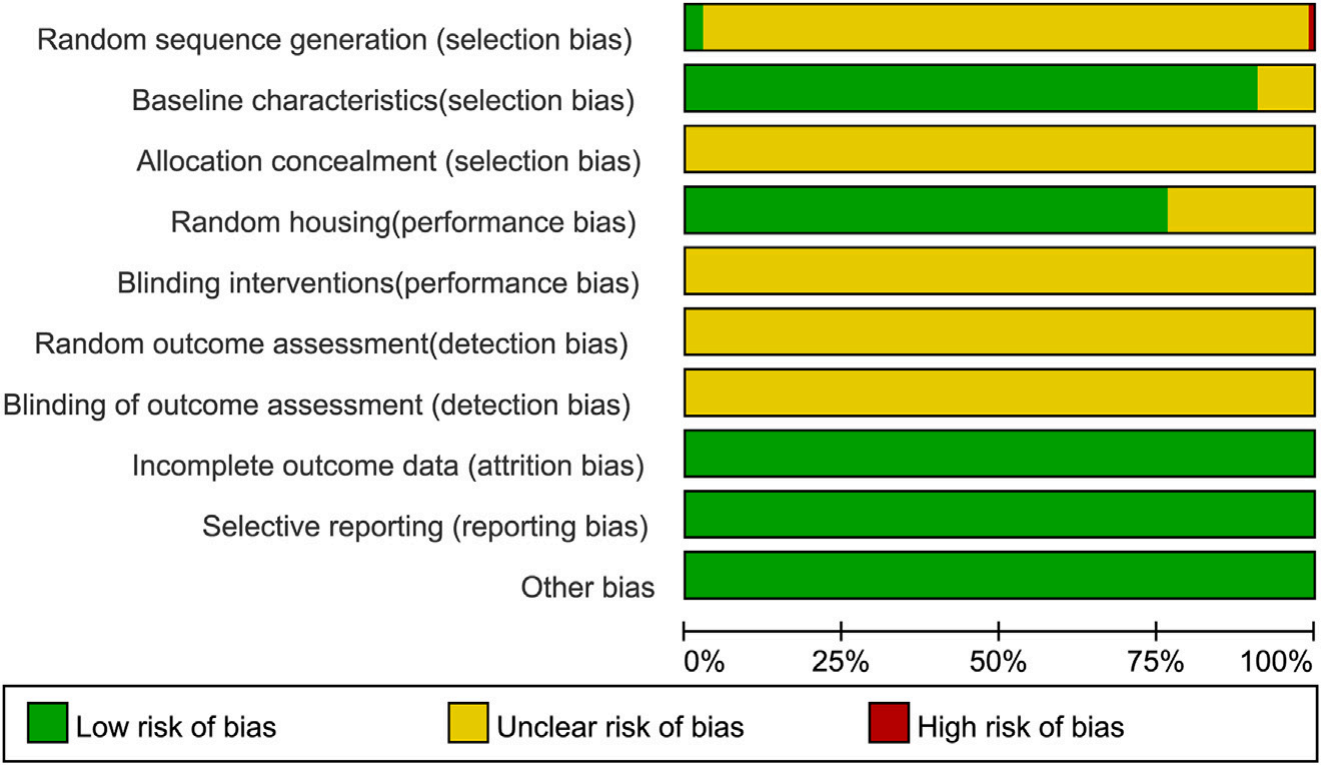
Risk of bias and quality evaluation

The quality of the included studies was assessed according to the criteria of SYRCLE’s risk of bias tool.^142^ The quality score of all the included studies ranged from 3 to 6. None of the studies specifically describe allocation concealment, blinding interventions, random outcome assessment, or blinding of outcome assessment, so they were estimated to have an unclear risk of bias. All included studies did not indicate incomplete outcome data, selective reporting, or other bias, so we estimated them to be low risk of bias. One study^33^ clearly indicated grouping based on weight. Three studies^26^^,^^42^^,^^56^ explicitly stated that a random number table method was used to group. The remaining studies only stated that they were randomly assigned but did not mention the specific randomization method. Baseline characteristics such as gender, age, and weight of the animals were fully described in 90.7% of the studies but were not adequately reported in 9.2% of the articles. 89 publications clearly described that all experimental animals were kept in the same conditions and environment, while the other 30 studies did not provide sufficient relevant information. The results of the quality evaluation are presented in Figure 2 and Table S2.

### Observation indicators

#### Atherosclerotic lesion area

##### Longitudinal plaque area

**Figure 3:**
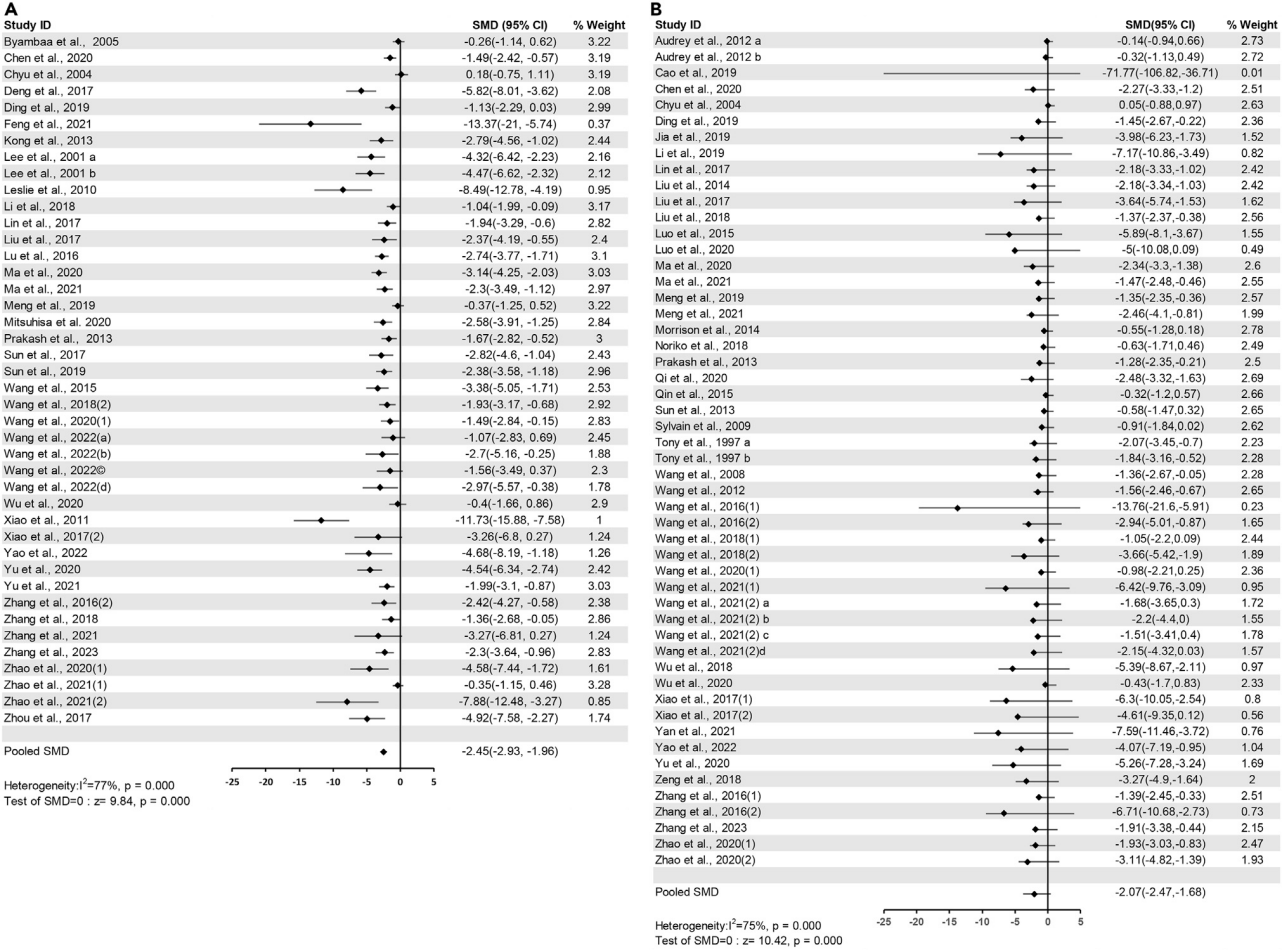
Forest plots of flavonoids effect on atherosclerotic lesion area (A) longitudinal plaque area; (B) cross-sectional plaque area.

The pooled effects of 42 comparisons from 38 studies indicated that the longitudinal plaque area significantly decreased in flavonoids groups compared with control groups (SMD = −2.45, 95%CI: −2.93 to −1.96, P<0.00001) (Figure 3A). Heterogeneity was significant in these studies (I^2^ = 77%, P<0.00001) (Table S3).

##### Cross-sectional plaque area

The pooled effects of 52 comparisons from 47 studies demonstrated that the cross-sectional plaque area significantly reduced in flavonoids groups compared with control groups (SMD = −2.07, 95%CI: −2.47 to −1.68, P<0.00001) (Figure 3B). Heterogeneity was notable (I^2^ = 75%, P<0.00001) (Table S3).

#### Serum lipid makers

##### TC

**Figure 4:**
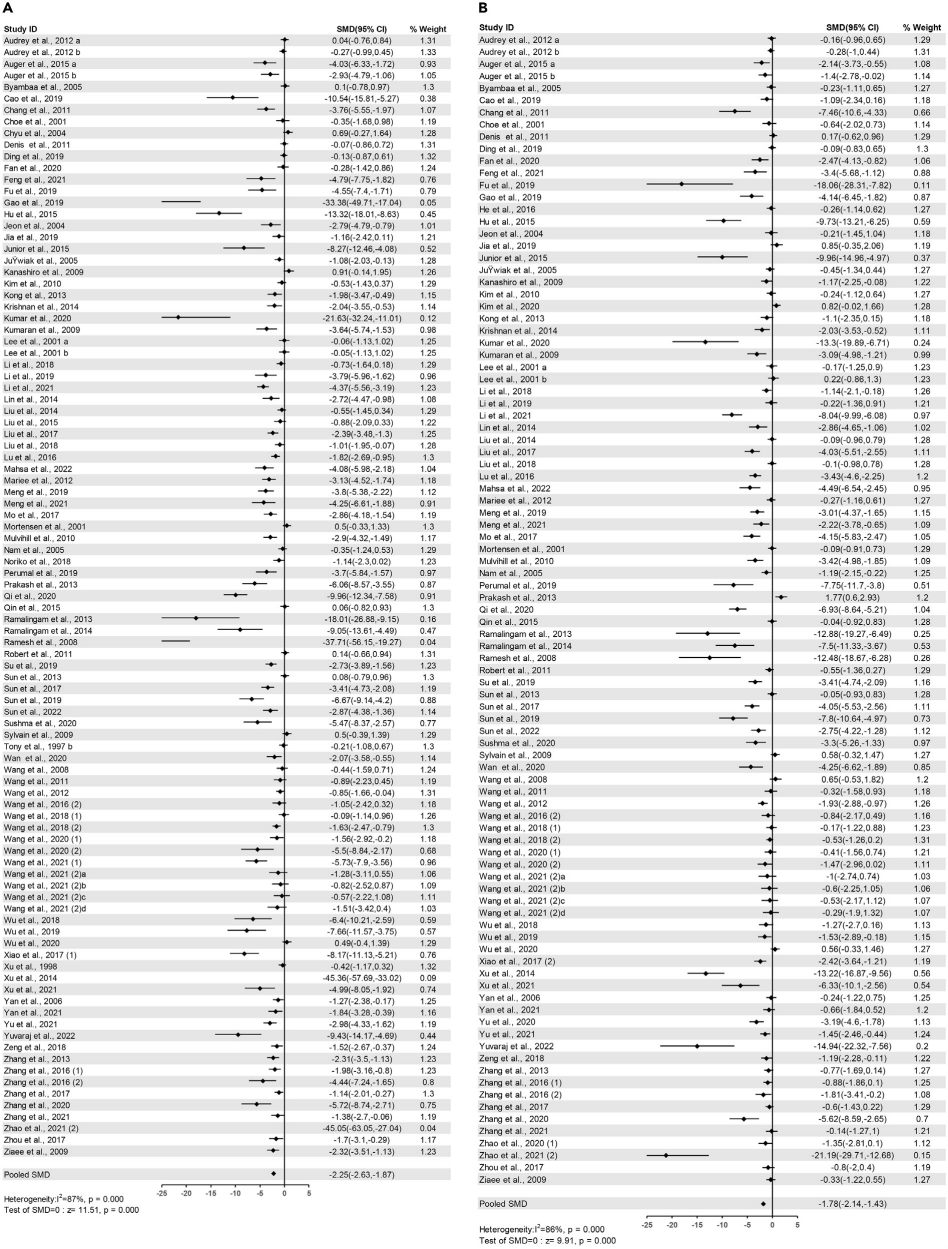
Forest plots of flavonoids effect on serum lipid markers (A) TC; (B) TG.

The pooled effects of 97 comparisons from 91 studies demonstrated that serum total cholesterol (TC) significantly reduced in flavonoids groups compared with control groups (SMD=-2.25, 95%CI: −2.63 to −1.87, P<0.00001) (Figure 4A). Heterogeneity was remarkable (I^2^ = 87%, P<0.00001) (Table S3).

##### TG

The pooled effects of 95 comparisons from 89 studies demonstrated that serum TG significantly decreased in flavonoids groups compared with control groups (SMD=-1.78, 95%CI: −2.14 to −1.43, P<0.00001) (Figure 4B). Heterogeneity was statistically significant (I^2^ = 86%, P<0.00001) (Table S3).

##### LDL-C

**Figure 5:**
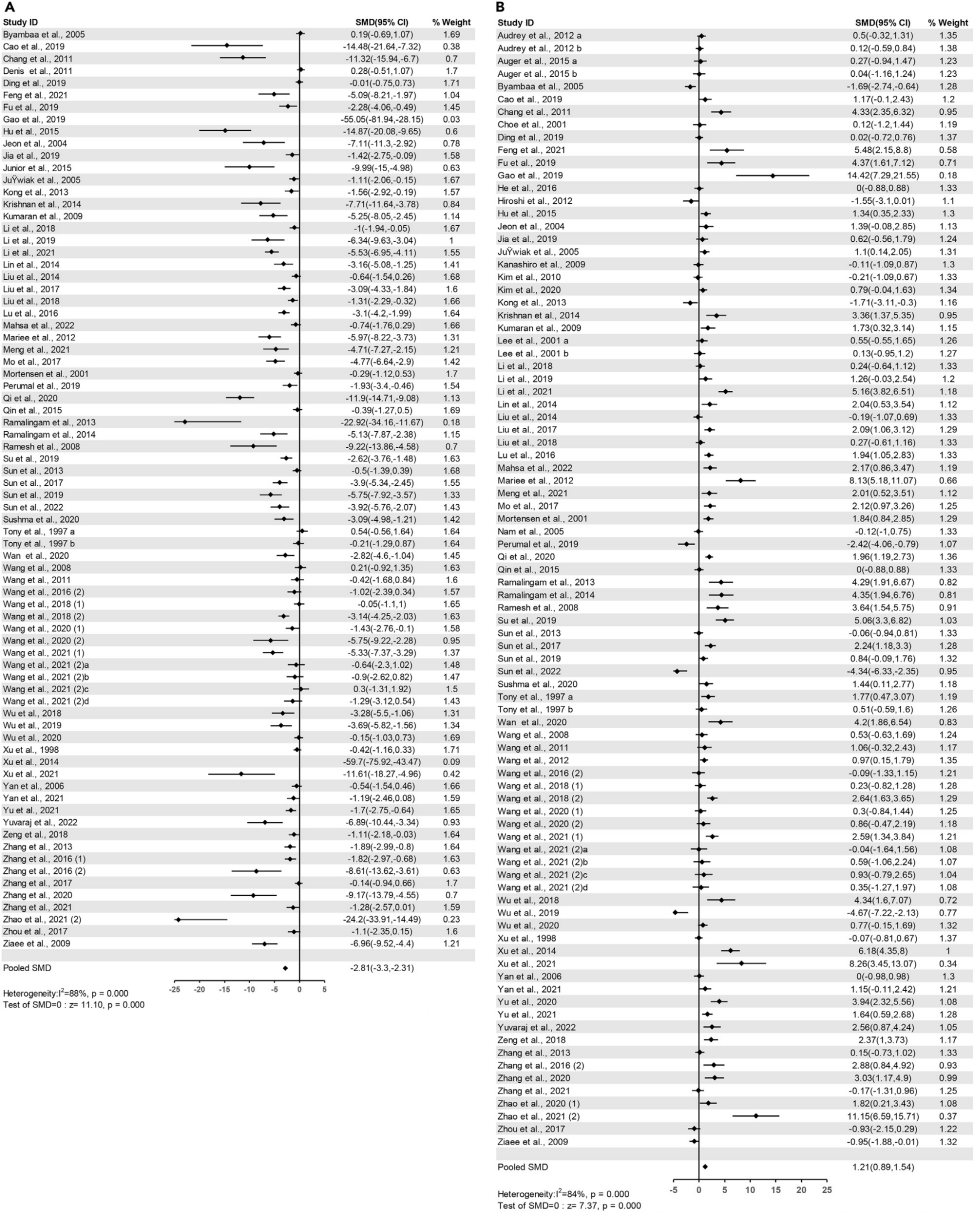
Forest plots of flavonoids effect on serum lipid markers (A) LDL-C; (B) HDL-C.

The pooled effects of 76 comparisons from 73 studies demonstrated that serum LDL-C significantly decreased in flavonoids groups compared with control groups (SMD=-2.81, 95%CI: −3.30 to −2.31, P<0.00001) (Figure 5A). Heterogeneity was statistically significant (I^2^ = 88%, P<0.00001) (Table S3).

##### HDL-C

The pooled effects of 88 comparisons from 81 studies demonstrated that serum HDL-C significantly increased in flavonoids groups compared with control groups (SMD=1.21, 95%CI: 0.89 to 1.54, P<0.00001) (Figure 5B). Heterogeneity was statistically significant (I^2^ = 84%, P<0.00001) (Table S3).

#### Circulating inflammatory factors

##### TNF-α

**Figure 6:**
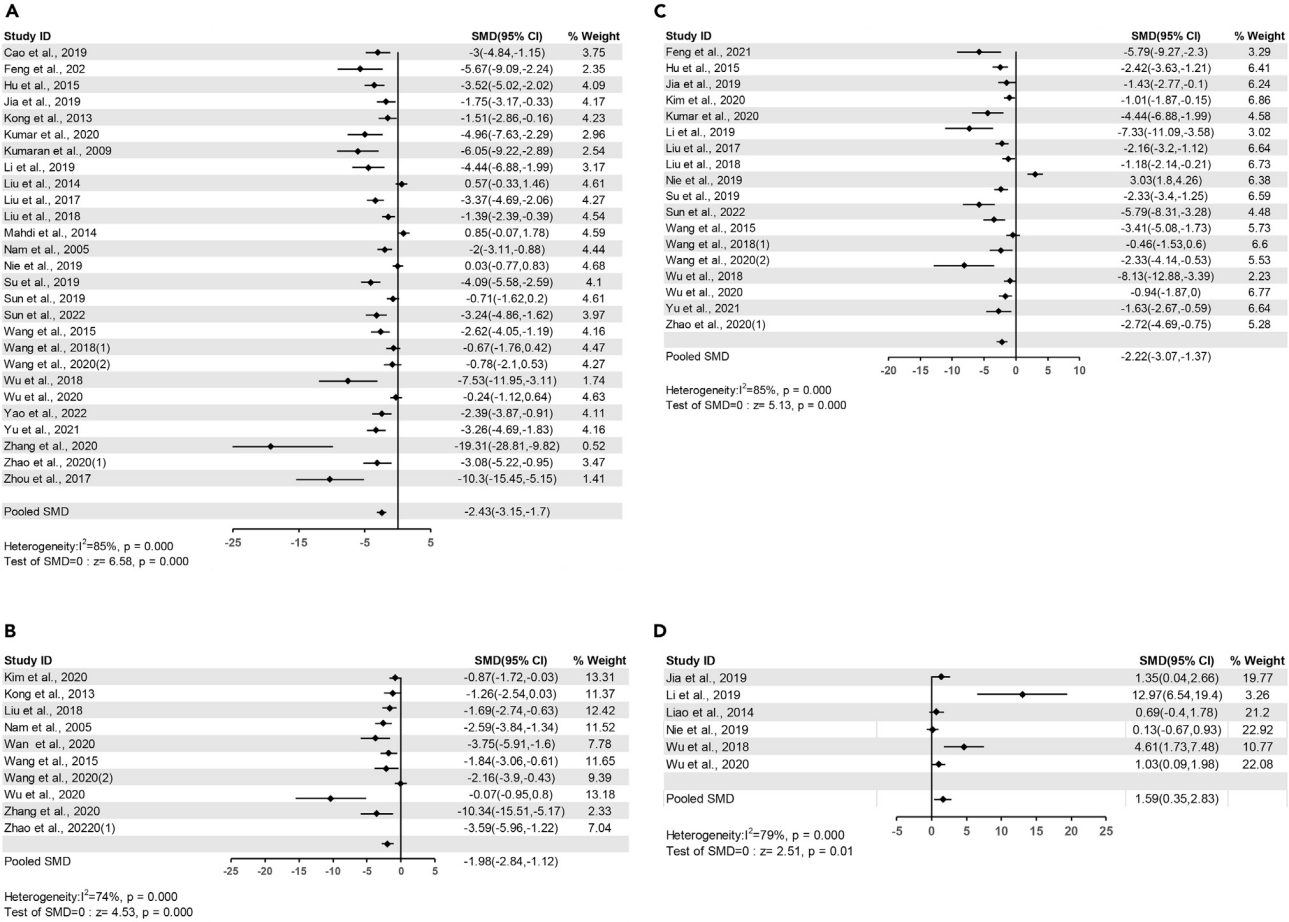
Forest plots of flavonoids effect on circulating inflammatory factors (A) TNF-α; (B) IL-1β; (C) IL-6; (D) IL-10.

The pooled effects of 27 comparisons demonstrated that flavonoids significantly decreased plasma TNF-αlevel (SMD=-2.43, 95%CI: −3.15 to −1.70, P<0.00001) (Figure 6A). Heterogeneity was statistically significant (I^2^ = 85%, P<0.00001) (Table S3).

##### IL-1β

The pooled effects of 10 comparisons demonstrated that flavonoids significantly decreased plasma IL-1β level (SMD=-1.98, 95%CI: −2.84 to −1.12, P<0.00001) (Figure 6B). Heterogeneity was statistically significant (I^2^ = 74%, P<0.0001) (Table S3).

##### IL-6

The pooled effects of 18 comparisons demonstrated that flavonoids significantly decreased plasma IL-6 level (SMD=-2.22, 95%CI: −3.07 to −1.37, P<0.00001) (Figure 6C). Heterogeneity was statistically significant (I^2^ = 85%, P<0.00001) (Table S3).

##### IL-10

The pooled effects of 6 comparisons demonstrated that flavonoids significantly increased plasma IL-10 level (SMD=1.59, 95%CI: 0.35 to 2.83, P=0.01) (Figure 6D). Heterogeneity was statistically considerable (I^2^ = 79%, P=0.0002) (Table S3).

### Potential molecular mechanisms

The potential molecular mechanisms of various flavonoids effects on atherosclerosis in eligible studies are summarized in Table S4.

### Subgroup analysis

The subgroup analysis was carried out to demonstrate the significant influencing factor of heterogeneity and determine the effect of the subclasses of flavonoids on the outcome measures. We found that the type of flavonoids may not be the potential source of heterogeneity (Table S3).

### Sensitivity analysis

**Figure 7:**
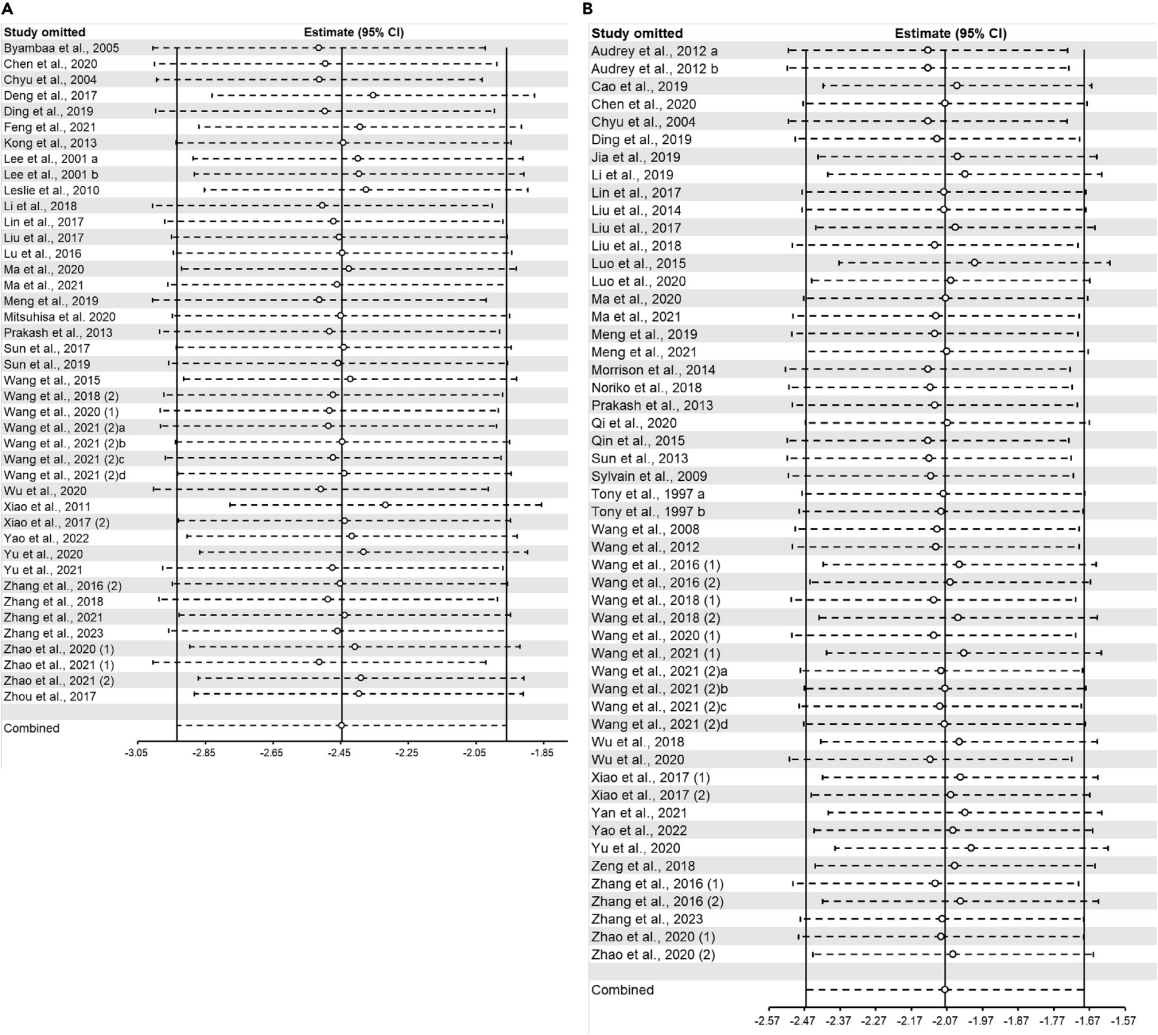
Sensitivity analysis of flavonoids effect on atherosclerotic lesion area (A) longitudinal plaque area; (B) cross-sectional plaque area.

**Figure 8:**
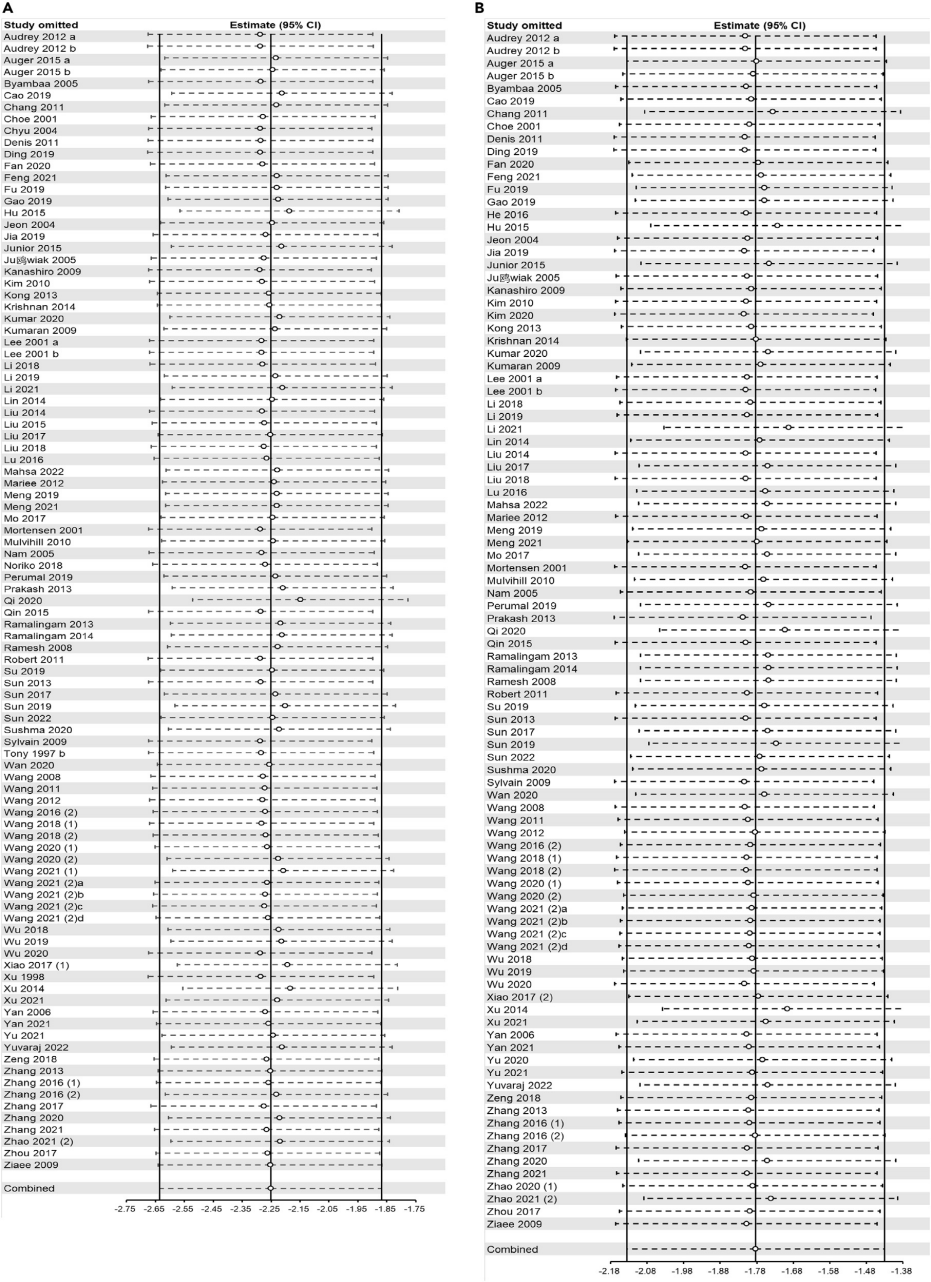
Sensitivity analysis of flavonoids effect on serum lipid markers (A) TC; (B) TG.

**Figure 9:**
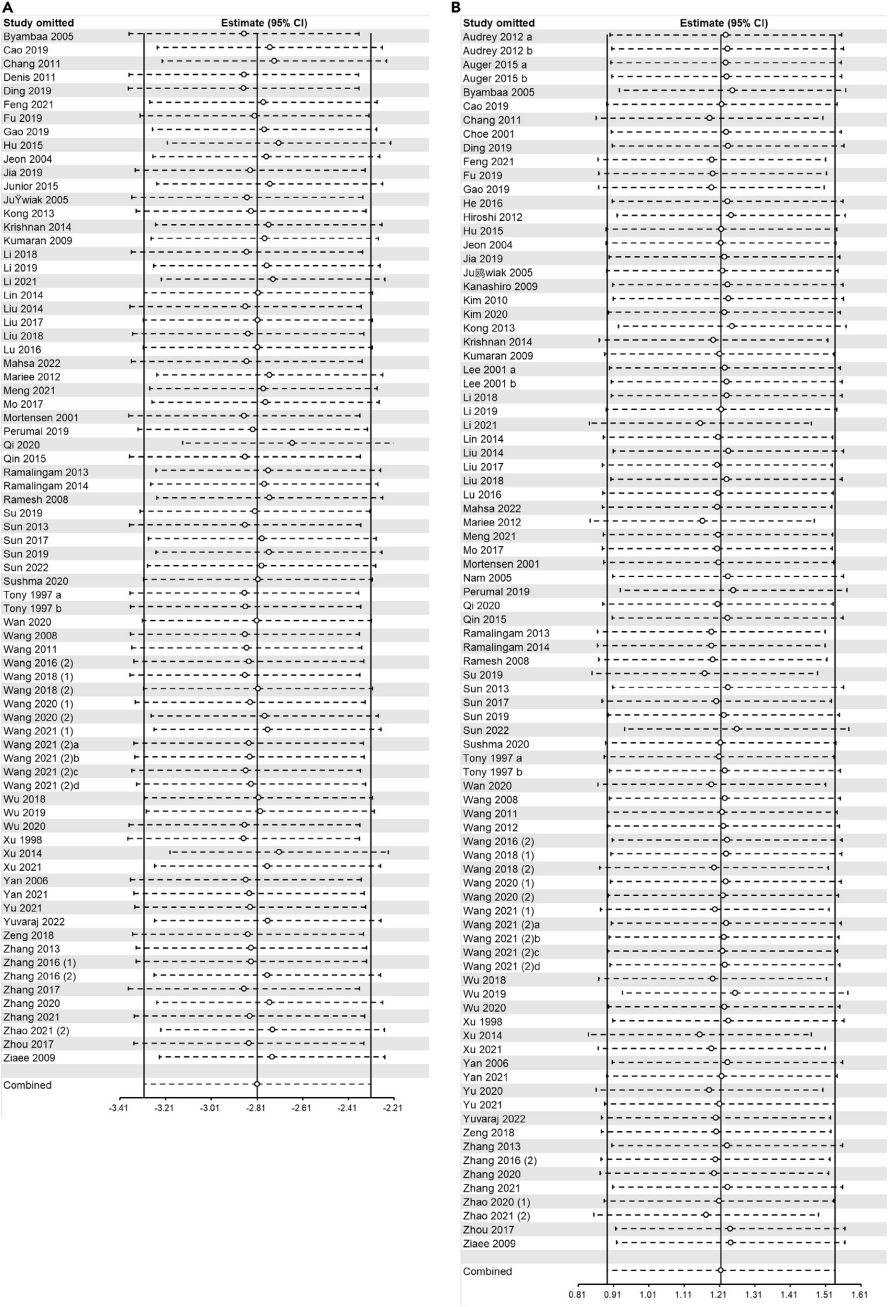
Sensitivity analysis of flavonoids effect on serum lipid markers (A) LDL-C; (B) HDL-C.

**Figure 10:**
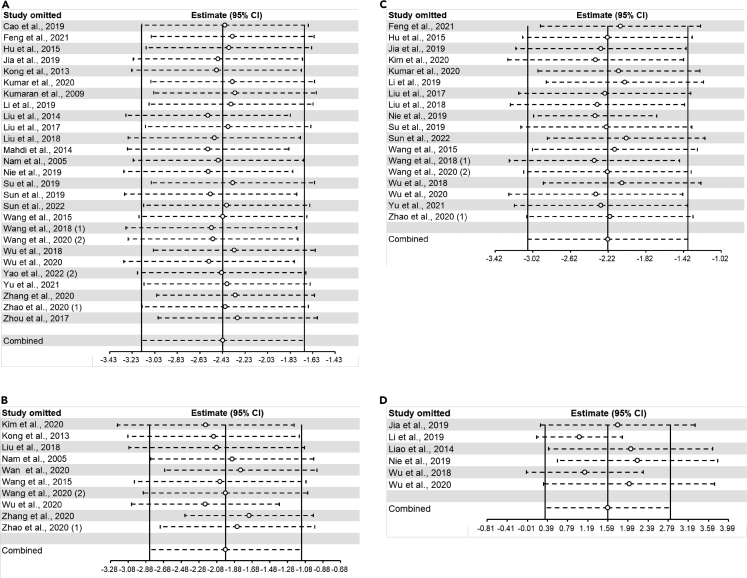
Sensitivity analysis of flavonoids effect on circulating inflammatory factors (A) TNF-α; (B) IL-1β; (C) IL-6; (D) IL-10.

For atherosclerotic lesion area (longitudinal and cross-sectional plaque area), serum lipid markers (TC, TG, LDL-C, and HDL-C), and circulating inflammatory factors (TNF-α, IL-1β, IL-6, and IL-10), sensitivity analysis was performed to confirm and account for the stability of the positive results. In summary, no statistically significant change in pooled effects was detected after excluding each study separately, which suggested the results remained robust. The results of the sensitivity analysis showed that the combined effects of all outcome measures except serum IL-10 were robust (Figures 7, 8, 9, and 10).

### Meta-regression analysis

Meta-regression analysis by subclass of flavonoids and species had no impact on the pooled effect size of all outcome measures (P>0.05) (Table S5).

### Publication bias

**Figure 11:**
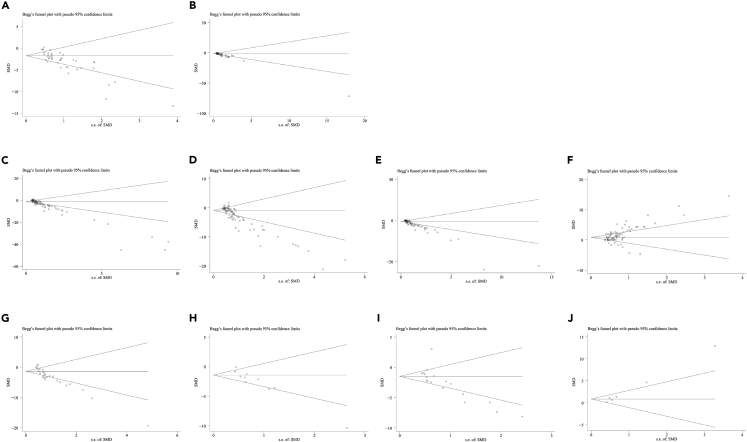
Begg’s test to assess publication bias (A) Longitudinal plaque area; (B) cross-sectional plaque area; (C) TC; (D) TG; (E) LDL-C; (F) HDL-C; (G) TNF-α; (H) IL-1β; (I) IL-6; (J) IL-10.

As shown in Figure 11 and Table S6, results from both Begg’s test and Egger’s test indicated significant publication bias for all outcome measures (P<0.05). However, further analysis with trim-and-fill computation demonstrated that publication bias did not change the pooled effect sizes (Figure S1).

## Discussion

Flavonoids are plant secondary metabolites widely found in nature.^13^ They maintain lipid homeostasis by regulating lipid metabolism, which is, increasing reverse cholesterol transport or HDL-C, or protecting against HDL dysfunction.^143^^,^^144^ Flavonoids also play important roles in inhibiting the onset and development of inflammatory diseases by exerting their powerful anti-inflammatory properties via inhibiting regulatory enzymes or transcription factors involved in inflammation.^145^ Furthermore, flavonoids can regulate apoptosis through the modulation of the levels of pro- and anti-apoptosis proteins.^146^ Accumulating evidence demonstrates that flavonoids can scavenge free radicals and inhibit metal-ion chelators to exert antioxidant effect.^147^ Additionally, flavonoids can improve the functions of endothelial (ECs) and vascular smooth muscle cells (VSMCs) by inhibiting or stimulating diverse ion channels.^148^ On account of their multiple biological activities, flavonoids have been applied to prevent and treat cancer and cardiovascular diseases.^149^ Atherosclerosis is a chronic lipid-driven inflammatory disease of large and medium-sized arteries.^2^^,^^5^ In atherosclerosis patients, anti-inflammatory therapy may reduce thrombotic risk,^150^ and maintaining optimal lipid levels can minimize the rate of progression of atherosclerotic plaques.^151^ Therefore, we presume that flavonoids can ameliorate atherosclerosis. In this study, a comprehensive meta-analysis of existing preclinical studies on the efficacy of flavonoids in atherosclerosis was performed. The results of the included studies demonstrated that flavonoids significantly decreased serum lipid levels, plasma pro-inflammatory factor concentrations, and atherosclerotic lesion area in animal models.

### Lipid-lowering effects and mechanisms of flavonoids

It is well known that hypercholesterolemia, one of the major risk factors for the occurrence and development of atherosclerosis, is one of the main therapeutic targets of atherosclerosis at present. Epidemiological data show that TG has a causal relationship with the development of atherosclerosis.^152^ There is substantial evidence that a high level of plasma LDL-C contributes to the progression of atherosclerosis.^153^ In contrast, the level of plasma HDL-C is negatively correlated with atherosclerosis.^154^ The reverse cholesterol transport process, an endogenous mechanism that allows cells to export cholesterol, is crucial for the maintenance of lipid homeostasis. ATP-binding cassette subfamily A member 1 (ABCA1) and G member 1 (ABCG1) are critical receptors for the initial and rate-limiting steps of reverse cholesterol transport and contribute to cholesterol efflux in foam cells.^155^^,^^156^ In the process, ABCA1 mediates the initial transport of cellular cholesterol to apolipoprotein A-I for forming nascent HDL particles, and ABCG1 promotes subsequent continued cholesterol efflux. Liver X receptors (LXR) regulate both ABCA1 and ABCG1, and facilitate reverse cholesterol transport.^157^ Studies have shown that flavonoids can promote cholesterol efflux by up-regulating the LXRα-ABCA1/ABCG1 pathway, thereby reducing foam cell formation.^38^^,^^56^^,^^64^^,^^124^^,^^136^

There is clear evidence that proprotein convertase subtilisin kexin type 9 (PCSK9) plays a crucial role in the maintenance of lipid homeostasis. PCSK9, a serine protease belonging to the proprotein convertase family, is mainly produced by the liver.^158^ LDL receptor (LDLR) is a key factor in the regulation of plasma cholesterol levels that clears circulating LDL-C via clathrin-mediated endocytosis.^159^ PCSK9 promotes the degradation of LDLR by targeting this receptor to the lysosome^160^ and inhibits LDLR recirculation to the cell surface.^161^ When the catalytic domain of PCSK9 binds to LDLR, the Cys-His-rich domain (CHRD) of PCSK9 interacts with cyclase-associated protein-1 (CAP-1) to lead the LDLR/PCSK9/CAP1 complex to lysosomal degradation through the caveolin-dependent pathway.^162^ In addition, PCSK9 inhibits ABCA1-mediated reverse cholesterol transport through downregulation of ABCA1.^163^ A preclinical investigation indicated that epigallocatechin gallate up-regulated LDLR expression by suppressing PCSK9 production, resulting in lowering LDL-C levels.^164^ Wang et al. found that naringin promoted reverse cholesterol transport by inhibiting PCSK9 in APOE^−/−^ mice.^108^ Studies have shown that quercetin accelerated cholesterol efflux by down-regulating PCSK9 to increase ABCA1 expression.^42^^,^^56^

### Anti-inflammatory effects and mechanisms of flavonoids

Lipid accumulation in macrophages induces inflammation, which in turn promotes and accelerates the development of atherosclerosis.^165^ Inflammation is involved in the initiation and development of atherosclerotic plaques.^166^ In the initial stage of atherosclerosis, LDL-C accumulate in the intima and activates the endothelium. Injured endothelial cells release chemokines and adhesion, attract monocytes to adhere to the endothelium, and then macrophages infiltrate into the subendothelium to form foam cells.^167^ Many cytokines, such as TNF-α, IL-1β, IL-6, and IL-10, are involved in this process. TNF-α, IL-1β, and IL-6 are pro-inflammatory cytokines, and the pro-inflammatory response mediated by them augments plaque growth and instability.^167^^,^^168^ IL-10 is an anti-inflammatory cytokine, and the major roles which IL-10 protects against atherogenesis include reduction of apoptosis, inhibition of pro-inflammatory cytokines, and regulation of lipid homeostasis.^169^ Toll-like receptor 4 (TLR4), a pattern recognition receptor for innate immunity, induces the production of pro-inflammatory cytokines through activating nuclear factor-κB (NF-κB).^170^ In addition, TLR4 negatively regulates ABCG1, which is also a key gene that mediates inflammation.^171^ NF-κB family are key regulators of inflammation in atherosclerosis.^172^ Moreover, NF-κB activation in macrophages mediates foam cell biogenesis.^173^ Heme oxygenase-1 (HO-1), encoded by the gene HMOX1 in humans,^174^ is the first and rate-limiting inducible enzyme of heme degradation^175^ and exhibits anti-inflammatory properties.^176^ The transcription factor NF-κB can bind the HMOX1 promoter to upregulate HO-1.^177^ Besides, HO-1 transcription is initiated by activated peroxisome proliferator-activated receptor γ (PPARγ), which is translocated to further activate PPAR response elements (PPREs).^178^ The silent information regulator sirtuin 1 (SIRT1) plays a role in anti-inflammation by inhibiting NF-κB signaling via deacetylating the p65 subunit of NF-κB complex.^179^ Moreover, SIRT1 activity is promoted by AMP-activated protein kinase (AMPK) via increasing intracellular NAD+ levels.^180^ Interestingly, SIRT1 is a target gene of PPAR-α and is suppressed by PPAR-α.^181^ Zhao et al. suggested that astragalin retards atherosclerosis by inhibiting the inflammatory response via down-regulating the TLR4/NF-κB pathway.^136^ Liu et al. demonstrated that kuwanon G improved inflammation by reducing the activity of NF-κB.^64^ Yu et al. found that biochanin A reduced inflammation by up-regulating the PPARγ/HO-1 pathway.^124^ Li et al. reported that luteolin prevented plaque development by decreasing macrophage inflammation via decreasing the AMPK-SIRT1 signaling.^55^

### Effects and mechanisms of flavonoids on reducing atherosclerotic plaque area

It is generally accepted that ECs, macrophages, and VSMCs play the predominant roles in the pathogenesis of atherosclerosis.^182^ When activated by stimuli related to cardiovascular risk factors, endothelial cells can secrete critical leukocyte adhesion molecules, such as vascular cell adhesion molecular-1 (VCAM-1), to promote circulating monocytes to the endothelial layer.^182^ Mediated by chemoattractant cytokines, macrophages migrate into the intima, and engulf the lipid, transforming into foam cells. Besides, VSMCs in the tunica media, in response to leukocyte mediators, migrate into the intima and give rise to foam cells. During the progression of atherosclerotic lesion, VSMCs produce extracellular matrix (such as interstitial collagen) that increases the thickness of the intimal layer. Furthermore, activated macrophages can increase the secretion of matrix metalloproteinases (MMPs) that degrade interstitial collagen.^183^ As the lesion advances, foam cells can undergo cell death, and the apoptotic cells release the lipid, which exacerbates inflammation and oxidative stress.^184^ A study showed that cyanidin-3-*O*-glucoside could attenuate endothelial cell dysfunction by inhibiting miR-204-5p/SIRT1-mediated inflammation and apoptosis.^109^ Chen et al. found that corylin could reduce the formation of atherosclerotic plaque by decreasing the production of VCAM-1 to inhibit monocyte adhesion via down-regulating the ROS/JNK pathway.^28^ Wang et al. reported that icariin could inhibit atherosclerosis progress through reducing macrophage infiltration via down-regulating the expression of CX3C chemokine receptor 1 (CX3CR1).^104^ Zhang et al. demonstrated that icariin contributed to plaque stabilization by facilitating collagen accumulation via inhibiting interstitial collagenase-1 (MMP-1).^130^ Wang et al. found that apple procyanidins significantly reduced aortic intimal-medial thickness on ultrasonography and the lipid accumulation area stained with Sudan IV in a rabbit model.^185^ A preclinical study demonstrated that quercetin decreased the aortic lesions by 20–70% based on ultrasound biomicroscopy analyses.^54^ Two observational studies suggested that pycnogenol reduced the arterial lesion progression that was evaluated using the ultrasonic arterial score based on the arterial wall morphology and the number of plaques that progressed.^54^^,^^186^^,^^187^

### Conclusions

In animal models, flavonoids decreased the levels of serum lipids and circulating pro-inflammatory factors, thereby reducing the atherosclerosis plaque size. In terms of molecular mechanisms, flavonoids improved lipid metabolism through various mechanisms, including the LXRα-ABCA1/ABCG1 pathway and the PCSK9-LDLR signaling, to exert anti-atherosclerosis functions. Moreover, flavonoids exhibited anti-atherosclerosis properties through anti-inflammatory mechanisms like the TLR4/NF-κB pathway and the AMPK-SIRT1 signaling. Besides, flavonoids reduced the atherosclerosis plaque size by diverse mechanisms, such as the ROS/JNK pathway and the CX3CR1 signaling, leading to anti-atherosclerosis effects. In summary, flavonoids exerted anti-atherosclerosis effects by reducing the levels of serum lipids and circulating pro-inflammatory factors and the size of atherosclerotic plaque, which provides evidence for the potential cardiovascular benefits of flavonoids.

### Limitations of the study

First, preclinical studies only suggest a potential for cardioprotection by flavonoids, and there is a need for clinical confirmation of these findings if they are to be considered valid. Second, considering the heterogeneity, the results must be explained circumspectly. The influence of flavonoid types and animal species on heterogeneity was basically excluded, but other possible influencing factors of heterogeneity, such as gender and age of animals and dose and duration of administration, were not fully analyzed due to insufficient information in the included literatures. In addition to the restrictive factors mentioned previously, there are still other unpredictable factors in the included studies that need to be further verified by more experiments. Third, the efficacy of different subclasses of flavonoids was not compared.

## STAR★Methods

### Key resources table

**Table:** 

REAGENT or RESOURCE	SOURCE	IDENTIFIER
**Deposited data**		

Studies For Meta-analysis	PubMed, Web of Science database, Embase, and Cochrane Library	The studies included are referenced in Data S1

**Software and algorithms**		

STATA software version 15.1	Downloaded STATA software	https://www.stata.com/products/
Review Manager software version 5.4	Downloaded Review Manager software	https://training.cochrane.org/online-learning/core-software/revman
Get Data Graph Digitizer software version 2.26	Downloaded Get Data Graph Digitizer software	http://getdata-graph-digitizer.com/download.php

### Resource availability

#### Lead contact

Further information and requests for resources should be directed to and will be fulfilled by the lead contact, Dongye Li (dongyeli@xzhmu.edu.cn).

#### Materials availability

This study did not generate new unique reagents.

#### Data and code availability

• The detailed search strategies are available in the supplemental information and listed in the key resources table. All data reported in this paper will be shared by the lead contact upon request.
• This paper does not report original code.
• Any additional information required to reanalyze the data reported in this paper is available from the lead contact upon request.

### Experimental model and study participant details

Our study does not use experimental models typical in the life sciences.

### Method details

#### Search Strategy

Relevant articles from August 1954 to April 2023 were systematically searched using PubMed, Web of Science database, Embase, and Cochrane Library. The specific search items included (“flavonoids” OR “flavonols” OR “flavones” OR “flavanols” OR “flavan-3-ols” OR “catechins” OR “flavanones” OR “anthocyanins” OR “isoflavones” OR “chalcones”) AND (“atherosclerosis” OR “atheroscleroses” OR “atherogenesis” OR “atherogeneses”). Additionally, the reference lists of included studies were also screened by two authors for relevant articles. The meta-analysis complied with the Preferred Reporting Items for Systematic Reviews and Meta-Analyses (PRISMA) guidelines.^188^

#### Inclusion and exclusion criteria

##### Types of studies

All studies were limited to original researches, and non-original researches were excluded, such as reviews, editorials, comments, conference abstracts, correspondences, case reports, etc. *In-vitro* studies or clinical studies were also excluded.

##### Types of Participants

Atherosclerosis or hyperlipidemia model animals of any species, age, and gender were all included. In addition to the above, other disease model animals were excluded.

##### Types of interventions

The intervention group was treated with flavonoid monomer alone, and the dose, duration, and route of administration were not limited. The control group was treated with vehicle (e.g., saline) or no treatment. Studies in combination with other drugs, or studies using extracts or polymers of flavonoids were excluded. Studies with a missing control group were excluded.

##### Types of observation indicators

The outcome observation indicators of this meta-analysis were the quantitative analysis of atherosclerotic lesion area, serum lipid markers, and circulating inflammatory factors. Hematoxylin-eosin (HE), Oil Red O, or Sudan IV staining of the whole aorta or the presentative part of the aorta, such as aortic root and aortic sinus, were acceptable, while studies without the available data on atherosclerotic lesion area were eliminated. Quantitative detection of serum total cholesterol (TC), triglyceride (TG), LDL-C, and high-density lipoprotein cholesterol (HDL-C) represented the lipid levels. The concentrations of serum tumor necrosis factor-α (TNF-α), interleukin-1β (IL-1β), interleukin-6 (IL-6), and interleukin-10 (IL-10) represented the inflammation levels in blood. Studies that were duplicated or could not be obtained with full texts or extracted data were removed.

#### Data extraction

The following categories were summarized as the baseline characteristics of the studies: (1) basic information: first author's surname, year of publication; (2) animal information: gene modification, gender, age, and weight of animals in each study; (3) intervention information: type of flavonoids, route, dose, and duration of administration; (4) outcome indexes: quantitative data on atherosclerotic lesion area (longitudinal and cross-sectional plaque area), serum lipid markers (TC, TG, LDL-C, HDL-C), and circulating inflammatory factors (TNF-α, IL-1β, IL-6, IL-10).

All the data on outcomes was continuous. The mean values, variance (standard deviation (SD) or standard error of mean (SEM)) and sample size of the control and intervention groups were extracted. SEM was converted to SD by utilizing the formula (SD = SEM ×√N). In case the outcomes were indispensable but only presented graphically, Get Data Graph Digitizer software version 2.26 was applied to quantify the results.^189^^,^^190^ When the flavonoid dosage or duration varied among the intervention groups, the group using the highest dose or the longest duration was recorded.^191^ The extraction of data from eligible articles was assessed by two independent authors, and disputes were resolved by discussion with the corresponding author.

#### Quality assessment

Two individual authors assessed the risk of bias of all included studies by using SYRCLE's risk of bias tool.^142^ The detailed criteria include: (1) random sequence generation; (2) baseline characteristics; (3) allocation concealment; (4) random housing; (5) blinding interventions; (6) random outcome assessment; (7) blinding of outcome assessment; (8) incomplete outcome data; (9) selective reporting; (10) other bias. Any dispute was decided by the corresponding author.

### Quantification and statistical analysis

Stata version 14 and Review Manager version 5.4 were adopted for data analysis and visualization. Due to differences in units or testing methods, the extracted outcomes were converted to the standardized mean difference (SMD) with the 95% confidence interval (CI) to reflect the pooled effect sizes. Statistical heterogeneity was calculated by using the I^2^ and Q statistic. Random-effects (RE) models were performed because of prevalent statistical heterogeneity (P<0.05, I^2^ > 50%) in preclinical studies. Post hoc subgroup analysis, sensitivity analysis, and meta-regression analysis were conducted to explore the sources of heterogeneity. The type of flavonoids (flavonols, flavones, flavanols (flavan-3-ols or catechins), flavanones, anthocyanidins, isoflavones, and chalcones) was considered as the latent subgroup basis. Leave-one sensitivity analysis was performed to estimate the robustness of the results, which was achieved by removing one study sequentially and repeating the meta-analysis. Besides, publication bias was evaluated with the Begg’s test, Egger’s test, and trim-and-fill method. P < 0.05 was considered statistically significant.

### Additional resources

Our study has not generated or contributed to a new website and it is not part of a clinical trial.

## Supplemental information

Document S1. Figure S1, Tables S5 and S6, and Data S1

Table S1. Basic characteristics of the included studies, related to STAR Methods

Table S2. Quality assessment of included studies, related to Figure 2

Table S3. Subgroup analysis based on the subclass of flavonoids, related to Figures 3, 4, 5, and 6

Table S4. Possible mechanisms of flavonoids on AS animal models, related to STAR Methods
