# Supplementary material for: Beneficial effects of flavonoids on animal models of atherosclerosis: A systematic review and meta-analysis
Source: iScience. 2023 Oct 27;26(11):108337. doi: 10.1016/j.isci.2023.108337 (PMC10665821; doi:10.1016/j.isci.2023.108337)
Supplement: Document S1. Figure S1, Tables S5 and S6, and Data S1 [file mmc1.pdf]

iScience, Volume *26*

Supplemental information

Beneficial effects of flavonoids on animal models of atherosclerosis: A systematic review and meta-analysis

Gege Liao, Wanlu Liu, Yiming Dai, Xiangxiang Shi, Yang Liu, Dongye Li, and Tongda Xu

Data S1. Search strategy, Related to STAR Methods.

1.PUBMED

#1 (Atherosclerosis) OR (Atheroscleroses) OR (Atherogenesis) OR (Atherogeneses)

#2 (Anthocyanidins) OR (Anthocyanin) OR (Leucoanthocyanidins) OR (Anthocyanidins) OR (Anthocyanidin)

#3 Flavanol

#4 (flavan-3-ol) OR (3-flavanol (mixed isomers)) OR (3,4-dihydro-2-phenyl-2H-1-benzopyran-3-ol) OR (3-flavan-ol) OR (3-flavanol) OR (2H-1-benzopyran-3-ol, 3,4-dihydro-2-phenyl-)

#5 (Catechin) OR ((+)-Catechin) OR (Cianidanol) OR (Catechinic Acid) OR (Catechuic Acid) OR ((+)-Cyanidanol) OR (3,3',4',5,7-Flavanpentol) OR (Catergen) OR (Zyma) OR (Epicatechin) OR ((-)-Epicatechin) OR ((2R,3R)-2-(3,4-Dihydroxyphenyl)-3,5,7-chromanetriol) OR (2H-1-Benzopyran-3,5,7-triol, 2-(3,4-dihydroxyphenyl)-3,4-dihydro-, (2R-cis)-) OR (KB-53) OR (KB 53) OR (KB53) OR (Z 7300) OR ((+)-Cyanidanol-3) OR (Cyanidanol-3) OR (Cyanidanol 3)

#6 (Flavanones) OR (2-Phenyl-Benzopyran-4-Ones) OR (2 Phenyl Benzopyran 4 Ones)

#7 (Flavones) OR (2-Phenyl-2-Ene-Benzopyran-4-One Compounds)

#8 (Flavonols) OR (3-hydroxy-4-keto-Flavonoids) OR (3 hydroxy 4 keto Flavonoids) OR (2-Phenyl-3-Hydroxy-Benzopyran-4-Ones) OR (2 Phenyl 3 Hydroxy Benzopyran 4 Ones)

#9 (Isoflavones) OR (Isoflavone Derivatives) OR (Derivatives, Isoflavone) OR (Isoflavone Derivative) OR (Derivative, Isoflavone) OR (Isoflavone) OR (Homoisoflavones) OR (3-Benzylidene-4-Chromanone) OR (3 Benzylidene 4 Chromanone) OR (Homoisoflavone) OR (3-Benzylidene-4-Chromanones) OR (3 Benzylidene 4 Chromanones) OR (3-Benzylchroman-4-Ones) OR (3 Benzylchroman 4 Ones) OR (3-Benzylchroman-4-One) OR (3 Benzylchroman 4 One)

#10 (chalcone) OR (Benzalacetophenone) OR (Chalkone) OR (1,3-Diphenyl-2-Propen-1-One) OR (1,3 Diphenyl 2 Propen 1 One) OR (Benzylideneacetophenone)

#11 (flavonoids) OR (2-Phenyl-Chromenes) OR (2 Phenyl Chromenes) OR (2-Phenyl-Benzopyran) OR (2 Phenyl Benzopyran) OR (2-Phenyl-Benzopyrans) OR (2 Phenyl Benzopyrans) OR (2-Phenyl-Chromene) OR (2 Phenyl Chromene) OR (Flavonoid) OR (Bioflavonoids) OR (Bioflavonoid)

#1 AND (#2 OR #3 OR #4 OR #5 OR #6 OR #7 OR #8 OR #9 OR #10 OR #11)

2.Web of Science

#1 TS=(Atherosclerosis OR Atheroscleroses OR Atherogenesis OR Atherogeneses)

#2 TS=(Anthocyanidins OR Anthocyanin OR Leucoanthocyanidins OR Anthocyanidins OR Anthocyanidin)

#3 TS=(Flavanol)

#4 TS=(flavan-3-ol OR 3-flavanol (mixed isomers) OR 3,4-dihydro-2-phenyl-2H-1-benzopyran-3-ol OR 3-flavan-ol OR 3-flavanol OR 2H-1-benzopyran-3-ol, 3,4-dihydro-2-phenyl-)

#5 TS=(Catechin OR (+)-Catechin OR Cianidanol OR Catechinic Acid OR Catechuic Acid OR (+)-Cyanidanol OR 3,3',4',5,7-Flavanpentol OR Catergen OR Zyma OR Epicatechin OR (-)-Epicatechin OR (2R,3R)-2-(3,4-Dihydroxyphenyl)-3,5,7-chromanetriol OR 2H-1-Benzopyran-3,5,7-triol, 2-(3,4-dihydroxyphenyl)-3,4-dihydro-,(2R-cis)- OR KB-53 OR KB 53 OR KB53 OR Z 7300 OR (+)-Cyanidanol-3 OR Cyanidanol-3 OR Cyanidanol 3)

#6 TS=(Flavanones OR 2-Phenyl-Benzopyran-4-Ones OR 2 Phenyl Benzopyran 4 Ones)

#7 TS=(Flavones OR 2-Phenyl-2-Ene-Benzopyran-4-One Compounds)

#8 TS=(Flavonols OR 3-hydroxy-4-keto-Flavonoids OR 3 hydroxy 4 keto Flavonoids OR 2-Phenyl-3-Hydroxy-Benzopyran-4-Ones OR 2 Phenyl 3 Hydroxy Benzopyran 4 Ones)

#9 TS=(Isoflavones OR Isoflavone Derivatives OR Derivatives, Isoflavone OR Isoflavone Derivative OR Derivative, Isoflavone OR IsoflavoneOR Homoisoflavones OR 3-Benzylidene-4-Chromanone OR 3 Benzylidene 4 Chromanone OR Homoisoflavone OR 3-Benzylidene-4-Chromanones OR 3 Benzylidene 4 Chromanones OR 3-Benzylchroman-4-Ones OR 3 Benzylchroman 4 Ones OR 3-Benzylchroman-4-One OR 3 Benzylchroman 4 One)

#10 TS=(chalcone OR Benzalacetophenone OR Chalkone OR 1,3-Diphenyl-2-Propen-1-One OR 1,3 Diphenyl 2 Propen 1 One OR Benzylideneacetophenone)

#11 TS=(flavonoids OR 2-Phenyl-Chromenes OR 2 Phenyl Chromenes OR 2-Phenyl-Benzopyran OR 2 Phenyl Benzopyran OR 2-Phenyl-Benzopyrans OR 2 Phenyl Benzopyrans OR 2-Phenyl-Chromene OR 2 Phenyl Chromene OR Flavonoid OR Bioflavonoids OR Bioflavonoid)

#1 AND (#2 OR #3 OR #4 OR #5 OR #6 OR #7 OR #8 OR #9 OR #10 OR #11) and Animals (MeSH 主题词)

3.EMBASE

#1 ‘Atherosclerosis’ OR ‘Atheroscleroses’ OR ‘Atherogenesis’ OR ‘Atherogeneses’

#2 ‘Anthocyanidins’ OR ‘Anthocyanin’ OR ‘Leucoanthocyanidins’ OR ‘Anthocyanidins’ OR ‘Anthocyanidin’

#3 ‘Flavanol’

#4 ‘flavan-3-ol’ OR ‘3-flavanol (mixed isomers)’ OR ‘3,4-dihydro-2-phenyl-2H-1-benzopyran-3-ol’ OR ‘3-flavan-ol’ OR ‘3-flavanol’ OR ‘2H-1-benzopyran-3-ol, 3,4-dihydro-2-phenyl-’

#5 ‘Catechin’ OR ‘Cianidanol’ OR ‘Catechinic Acid’ OR ‘Catechuic Acid’ OR ‘Catergen’ OR ‘Zyma’ OR ‘Epicatechin’ OR ‘(2R,3R)-2-(3,4-Dihydroxyphenyl)-3,5,7-chromanetriol’ OR ‘2H-1-Benzopyran-3,5,7-triol, 2-(3,4-dihydroxyphenyl)-3,4-dihydro-,(2R-cis)-’ OR ‘KB-53’ OR ‘KB 53’ OR ‘KB53’ OR ‘Z 7300’ OR ‘(+)-Cyanidanol-3’ OR ‘Cyanidanol-3’ OR ‘Cyanidanol 3’

#6 ‘Flavanones’ OR ‘2-Phenyl-Benzopyran-4-Ones’ OR ‘2 Phenyl Benzopyran 4 Ones’

#7 ‘Flavones’ OR ‘2-Phenyl-2-Ene-Benzopyran-4-One Compounds’

#8 ‘Flavonols’ OR ‘3-hydroxy-4-keto-Flavonoids’ OR ‘3 hydroxy 4 keto Flavonoids’ OR ‘2-Phenyl-3-Hydroxy-Benzopyran-4-Ones’ OR ‘2 Phenyl 3 Hydroxy Benzopyran 4 Ones’

#9 ‘Isoflavones’ OR ‘Isoflavone Derivatives’ OR ‘Derivatives, Isoflavone OR Isoflavone Derivative’ OR ‘Derivative, Isoflavone’ OR ‘Isoflavone’ OR ‘Homoisoflavones’ OR ‘3-Benzylidene-4-Chromanone’ OR ‘3 Benzylidene 4 Chromanone’ OR ‘Homoisoflavone’ OR ‘3-Benzylidene-4-Chromanones’ OR ‘3 Benzylidene 4 Chromanones’ OR ‘3-Benzylchroman-4-Ones’ OR ‘3 Benzylchroman 4 Ones’ OR ‘3-Benzylchroman-4-One’ OR ‘3 Benzylchroman 4 One’

#10 ‘chalcone’ OR ‘Benzalacetophenone’ OR ‘Chalkone’ OR ‘1,3-Diphenyl-2-Propen-1-One’ OR ‘1,3 Diphenyl 2 Propen 1 One’ OR ‘Benzylideneacetophenone’

#11 ‘flavonoids’ OR ‘2-Phenyl-Chromenes’ OR ‘2 Phenyl Chromenes’ OR ‘2-Phenyl-Benzopyran’ OR ‘2 Phenyl Benzopyran’ OR ‘2-Phenyl-Benzopyrans’ OR ‘2 Phenyl Benzopyrans’ OR ‘2-Phenyl-Chromene’ OR ‘2 Phenyl Chromene’ OR ‘Flavonoid’ OR ‘Bioflavonoids’ OR ‘Bioflavonoid’

#1 AND (#2 OR #3 OR #4 OR #5 OR #6 OR #7 OR #8 OR #9 OR #10 OR #11) AND [animal]/lim

4.Cochrane

#1 “Atherosclerosis” OR “Atheroscleroses” OR “Atherogenesis” OR “Atherogeneses”

#2 “Anthocyanidins” OR “Anthocyanin” OR “Leucoanthocyanidins” OR “Anthocyanidins” OR “Anthocyanidin”

#3 Flavanol

#4 “flavan-3-ol” OR “3-flavanol (mixed isomers)” OR “3,4-dihydro-2-phenyl-2H-1-benzopyran-3-ol” OR “3-flavan-ol” OR “3-flavanol” OR “2H-1-benzopyran-3-ol, 3,4-dihydro-2-phenyl-”

#5 “Catechin” OR “(+)-Catechin” OR “Cianidanol” OR “Catechinic Acid” OR “Catechuic Acid” OR “(+)-Cyanidanol” OR “3,3',4',5,7-Flavanpentol” OR “Catergen” OR “Zyma” OR “Epicatechin” OR “(-)-Epicatechin” OR “(2R,3R)-2-(3,4-Dihydroxyphenyl)-3,5,7-chromanetriol” OR “2H-1-Benzopyran-3,5,7-triol, 2-(3,4-dihydroxyphenyl)-3,4-dihydro-, (2R-cis)-” OR “KB-53” OR “KB 53” OR “KB53” OR “Z 7300” OR “(+)-Cyanidanol-3” OR “Cyanidanol-3” OR “Cyanidanol 3”

#6 “Flavanones” OR “2-Phenyl-Benzopyran-4-Ones” OR “2 Phenyl Benzopyran 4 Ones”

#7 “Flavones” OR “2-Phenyl-2-Ene-Benzopyran-4-One Compounds”

#8 “Flavonols” OR “3-hydroxy-4-keto-Flavonoids” OR “3 hydroxy 4 keto Flavonoids” OR “2-Phenyl-3-Hydroxy-Benzopyran-4-Ones” OR “2 Phenyl 3 Hydroxy Benzopyran 4 Ones”

#9 “Isoflavones” OR “Isoflavone Derivatives” OR “Derivatives, Isoflavone” OR “Isoflavone Derivative” OR “Derivative, Isoflavone” OR “Isoflavone” OR “Homoisoflavones” OR “3-Benzylidene-4-Chromanone” OR “3 Benzylidene 4 Chromanone” OR “Homoisoflavone” OR “3-Benzylidene-4-Chromanones” OR “3 Benzylidene 4 Chromanones” OR “3-Benzylchroman-4-Ones” OR “3 Benzylchroman 4 Ones” OR “3-Benzylchroman-4-One” OR “3 Benzylchroman 4 One”

#10 “chalcone” OR “Benzalacetophenone” OR “Chalkone” OR “1,3-Diphenyl-2-Propen-1-One” OR “1,3 Diphenyl 2 Propen 1 One” OR “Benzylideneacetophenone”

#11 “flavonoids” OR “2-Phenyl-Chromenes” OR “2 Phenyl Chromenes” OR “2-Phenyl-Benzopyran” OR “2 Phenyl Benzopyran” OR “2-Phenyl-Benzopyrans” OR “2 Phenyl Benzopyrans” OR “2-Phenyl-Chromene” OR “2 Phenyl Chromene” OR “Flavonoid” OR “Bioflavonoids” OR “Bioflavonoid”

#1 AND (#2 OR #3 OR #4 OR #5 OR #6 OR #7 OR #8 OR #9 OR #10 OR #11)

Table S5. Results of meta-regression analysis, Related to Figure 3, 4, 5 and 6.

	Outcome mesures	Comparisons (studies)	Subclass of flavonoids		Species	
			t	p_t	t	p_t
Atherosclerotic lesion area	Longitudinal	42 (38)	-0.97	0.334	-1.48	0.139
	Cross-sectional	52 (47)	1.40	0.161	0.77	0.439
Serum lipid markers	TC	97 (91)	-0.06	0.948	0.43	0.670
	TG	95 (89)	-0.18	0.860	-0.46	0.647
	LDL-C	76 (73)	-0.03	0.974	-1.06	0.289
	HDL-C	88 (81)	-0.84	0.403	-0.96	0.338
Circulating inflammatory factors	TNF-α	27 (27)	-0.08	0.936	-0.60	0.549
	IL-1β	10 (10)	-1.17	0.240	0.01	0.992
	IL-6	18 (18)	-0.03	0.976	-0.83	0.409
	IL-10	6 (6)	-0.46	0.649	dropped	

HDL-C: high-density lipoprotein cholesterol; IL: interleukin; LDL-C: low-density lipoprotein cholesterol; TC: total cholesterol; TG: triglyceride; TNF: tumor necrosis factor.

Table S6. Begg's and Egger's test evaluating publication bias for included studies, Related to Figure 11.

	Outcome mesures	Comparisons (studies)	Begg's test		Egger's test	
			Z[a]	p_Z[a]	*t*	p_t
Atherosclerotic lesion area	Longitudinal	42 (38)	4.70	< 0.001	-7.69	< 0.001
	Cross-sectional	52 (47)	6.04	< 0.001	-9.33	< 0.001
Serum lipid markers	TC	97 (91)	9.38	< 0.001	-15.00	< 0.001
	TG	95 (89)	8.83	< 0.001	-12.63	< 0.001
	LDL-C	76 (73)	8.25	< 0.001	-13.61	< 0.001
	HDL-C	88 (81)	5.20	< 0.001	5.07	< 0.001
Circulating inflammatory factors	TNF-α	27 (27)	4.84	< 0.001	-8.22	< 0.001
	IL-1β	10 (10)	2.68	0.007	-5.58	0.001
	IL-6	18 (18)	3.41	0.001	-3.10	0.007
	IL-10	6 (6)	2.25	0.024	8.93	0.001

[a] Both Z and p_Z values were continually corrected.

HDL-C: high-density lipoprotein cholesterol; IL: interleukin; LDL-C: low-density lipoprotein cholesterol; TC: total cholesterol; TG: triglyceride; TNF: tumor necrosis factor.

Figure S1. The results of trim and fill computation, Related to Figure 11.

A. Longitudinal Plaque Area; B. Cross-sectional Plaque Area; C. TC; D. TG; E. LDL-C; F. HDL-C; G. TNF-α; H. IL-1β; I. IL-6; J. IL-10.

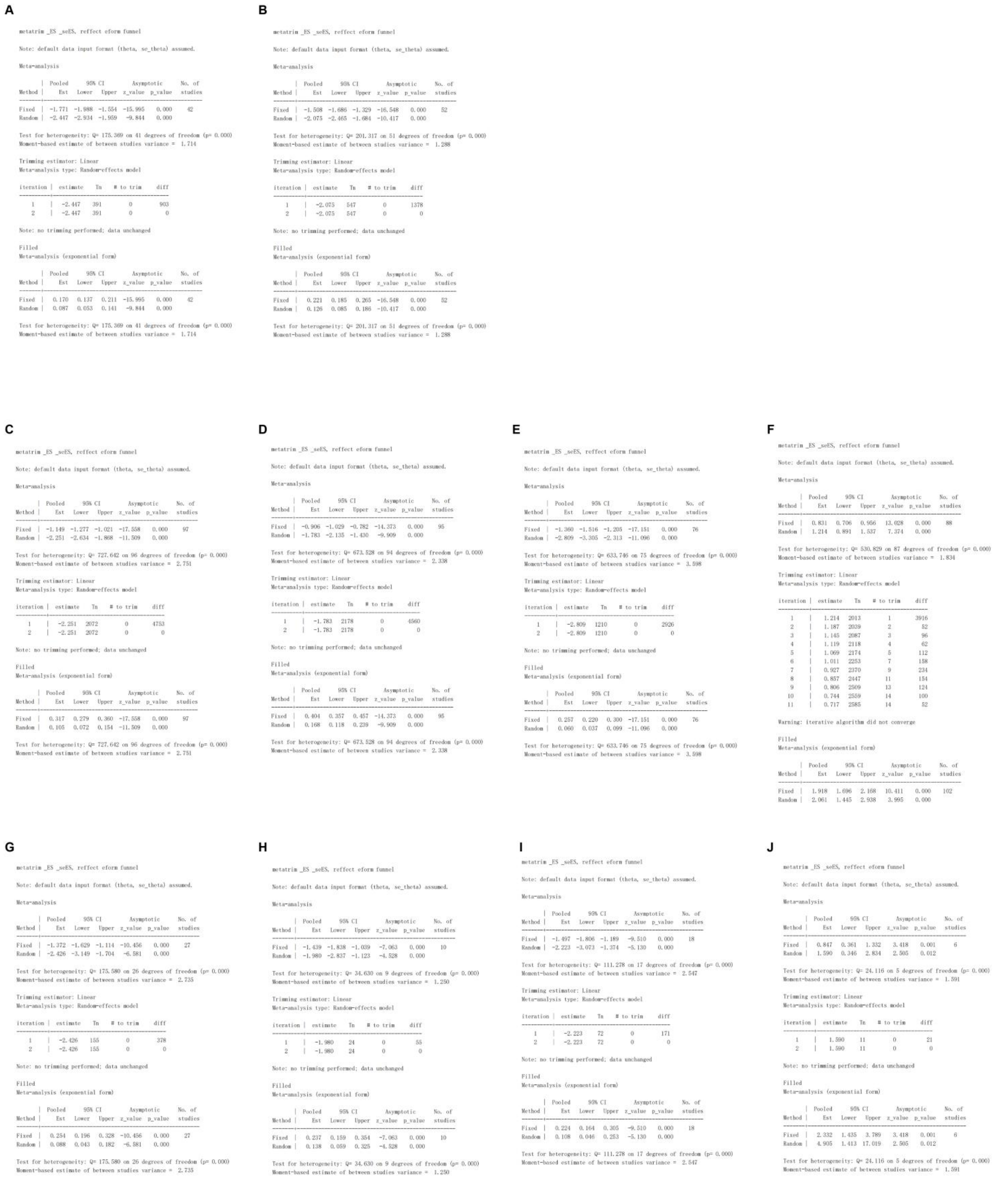

HDL-C: high-density lipoprotein cholesterol; IL: interleukin; LDL-C: low-density lipoprotein cholesterol; TC: total cholesterol; TG: triglyceride; TNF: tumor necrosis factor.
